# Game Fun Prediction Based on Frequency Domain Physiological Signals: Observational Study

**DOI:** 10.3390/s23167051

**Published:** 2023-08-09

**Authors:** Yeong-Yuh Xu, Chi-Huang Shih, Yan-Ting You

**Affiliations:** 1 Department of Artificial Intelligence and Computer Engineering, National Chin-Yi University of Technology, Taichung 411, Taiwan; yyxu@ncut.edu.tw; 2 Department of Computer Science and Information Engineering, National Chin-Yi University of Technology, Taichung 411, Taiwan; whale880808@gmail.com

**Keywords:** video game, photoplethysmography, heart rate variability

## Abstract

Traditionally, the subjective questionnaire collected from game players is regarded as a primary tool to evaluate a video game. However, the subjective evaluation result may vary due to individual differences, and it is not easy to provide real-time feedback to optimize the user experience. This paper aims to develop an objective game fun prediction system. In this system, the wearables with photoplethysmography (PPG) sensors continuously measure the heartbeat signals of game players, and the frequency domain heart rate variability (HRV) parameters can be derived from the inter-beat interval (IBI) sequence. Frequency domain HRV parameters, such as low frequency(LF), high frequency(HF), and LF/HF ratio, highly correlate with the human’s emotion and mental status. Most existing works on emotion measurement during a game adopt time domain physiological signals such as heart rate and facial electromyography (EMG). Time domain signals can be easily interfered with by noises and environmental effects. The main contributions of this paper include (1) regarding the curve transition and standard deviation of LF/HF ratio as the objective game fun indicators and (2) proposing a linear model using objective indicators for game fun score prediction. The self-built dataset in this study involves ten healthy participants, comprising 36 samples. According to the analytical results, the linear model’s mean absolute error (MAE) was 4.16%, and the root mean square error (RMSE) was 5.07%. While integrating this prediction model with wearable-based HRV measurements, the proposed system can provide a solution to improve the user experience of video games.

## 1. Introduction

As video games become prevailing worldwide, one of the main topics in game design and development is to collect feedback associated with the game content from the players. The players’ feedbacks build a connection between the game company and the users [1]. The game company can explore user preferences and evaluations to optimize the content design before publishing a new game. After the game is released, customers consider the feedback an essential reference to their purchasing intentions. On the other hand, real-time feedback during a game can be viewed as one of the game inputs for a better user experience. Traditionally, the questionnaire is a standard tool to collect subjective user feedback, for instance, the five-star rating. However, questionnaire-based feedback is easily affected by individual differences and may not fit real-time applications. In order to achieve an objective and real-time measurement of user experience, biofeedback sensors can be considered to gather physiological signals of the player during the game [2,3]. Typical biofeedback sensors include electrocardiography (ECG), electromyography (EMG), electrodermal activity (EDA), electrooculography (EOG), and electroencephalography (EEG).

In the domain of psychology, Csikszentmihalyi et al. propose the flow theory to describe a state of optimal user experience characterized by the following factors: matched challenge and skills; clear goals; immediate feedback; mind and hand; concentration on the task; a sense of potential control; the loss of self-consciousness; altered sense of time; and the autotelic experience [4,5]. While bringing the flow theory into video games, the matched challenge and skills indicate that a game player can experience the flow state more easily. The game player usually feels anxious when the skill cannot match the challenge. On the other hand, boredom occurs when the player’s skill easily conquers the challenge in the game. The game player could also become apathetic to the low skill and challenge. The core idea in designing a game is to avoid anxiety and boredom so that the game player can attain a higher level of game fun [6]. In the case that a game player stays in the apathetic state for a long time, the player can be far from game immersion. Smith et al. indicate that an appropriate level design is helpful for the players to achieve the flow state by raising their confidence in the game [7]. In [8], Schell et al. regard the game interest curve as an entertainment experience model. Typically, the interest curve fluctuates owing to the varied content during the game. Then, the highest interest peak is observed at some point, and the player attains an optimal experience.

The flow state and interest curve are featured with abstraction, subjectivity, and individual differences. Accordingly, building a connection between subjective flow experience and objective physiological signals becomes an essential challenge in the video game industry. In [2,9], it is shown that ECG and facial EMG signals can correspond to intense emotions (e.g., excitement and thrill) in a game. Specifically, the heart rate (HR) derived from ECG signals correlates with intense emotions up to 56% [2]. Cowley et al. indicate that for serious games, the inter-beat interval (IBI) can be associated with the studying performance [10]. Although physiological signals are effective biofeedback indicators for game players, a few works study the association of game flow state with physiological signals.

This paper aims to observe the flow state of game players based on heart rate variability (HRV) signals using photoplethysmography (PPG) sensors. PPG adopts a light source and a photodetector at the surface of the user’s skin to measure the volumetric variations of blood circulation in a non-invasive manner [11]. HRV indicates the variability of inter-beat intervals. Specifically, HRV corresponds to a complex interaction process by the autonomic nervous system involving the sympathetic nervous system (SNS), parasympathetic nervous system (PNS), and vagus nerve [12]. HRV signals can be analyzed in the time domain and frequency domain. Typical time domain HRV parameters are HR and the standard deviation of IBI. Frequency domain measurements divide the frequency bands into a low frequency (LF) ranging between 0.05 and 0.15 Hz and a high frequency (HF) ranging between 0.14 and 0.4 Hz. The ratio of LF-to-HF power (LF/HF) estimates the relative activity between the SNS and PNS under controlled conditions [13]. Reviewing the literature, HRV is shown to correlate with human emotions [14,15] and has connections with mental statuses such as stress [16,17], depression, and anxiety [18]. Traditionally, HRV-related parameters are derived from ECG signals. Recently, wearable devices with PPG sensors have been considered an alternative HRV measurement approach [19,20,21]. A previous study compared the pulse rate variability obtained from PPG with HRV information collected from a reference ECG device and found significant correlations of more than 82% for both time and frequency parameters [22]. Since PPG sensors are widely deployed in wearables to satisfy the requirements of daily use and mobility, PPG-based wearables have become useful tools for monitoring HRV changes.

In order to study the relationship between HRV and flow state, this paper first establishes a dataset comprising objective and subjective information collected from game players. In collecting objective physiological data, the PPG-based smart band measures the heartbeat signals of the participant during the game, and the corresponding HRV parameters are calculated. Most previous studies use time domain parameters such as HR and IBI [2,9,10,23]. This study considers the frequency domain parameter, namely LF/HF ratio, as the flow state indicator. The subjective data adopt the game fun scores given by participants immediately after the game. The analytical results show that the group with higher game fun scores has larger fluctuations in their LF/HF ratio curves. Based on the features extracted from the LF/HF ratio curve, a linear model is further proposed to predict game fun scores in an objective manner. The performance statistics indicate that the proposed linear prediction model can effectively monitor the flow state and assess the game fun degree.

This paper is organized as follows. Section 2 first describes the overview of a PPG-based HRV measurement system. Then, the materials and methods to collect the game fun dataset and their statistical results are reported. The proposed linear game fun score prediction is presented in Section 3. Section 4 gives the conclusions and future works.

## 2. Materials and Methods

### 2.1. PPG-Based HRV Measurement

**Figure 1:**
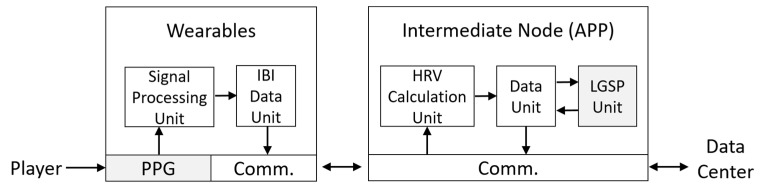
A wearable-based game fun score prediction system. Comm.: communication.

In this paper, the wearable device captures the user’s PPG signal during the game, and then, the collected time domain PPG signals are converted to frequency domain HRV parameters. Figure 1 shows the HRV measurement system using a PPG-based wearable device. In Figure 1, an intermediate node can communicate with the wearable device to start or stop PPG measurement. During the measurement, the wearable device continuously collects the PPG signal of the player. It performs noise reduction and baseline shift correction through the signal processing unit to ensure reliable signal quality. After collecting sufficient data within a period (e.g., 2 min), the inter-beat intervals derived from PPG data can be transmitted to the intermediate node via the communication interface. The intermediate node receives the IBI sequence, performs a fast Fourier transform (FFT) to obtain HRV parameters, and records them in the data unit. The HRV results can be stored in the data center later. In the next sub-section, the system mentioned above is utilized to conduct experiments in building a dataset of HRV data. According to the analytical results of the dataset in Section 3, the system shown in Figure 1 can further conduct the linear game fun score prediction (LGSP) based on the user’s HRV parameters.

**Figure 2:**
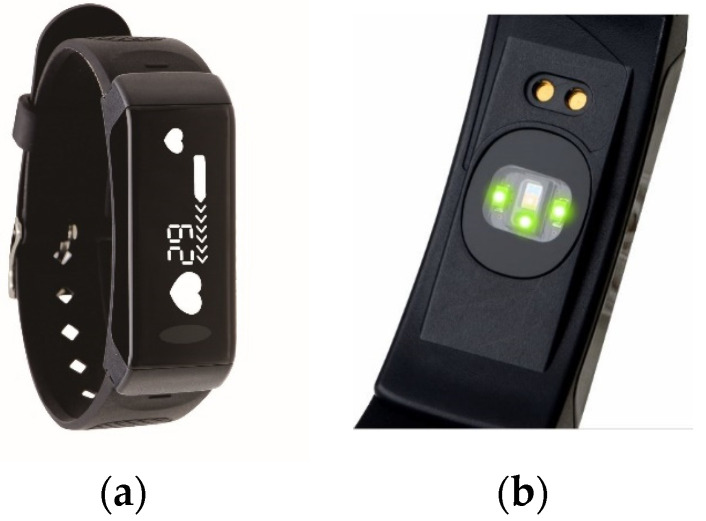
Wearable device for HRV measurement: (**a**) appearance on the front side; (**b**) three LEDs on the reverse side.

**Figure 3:**
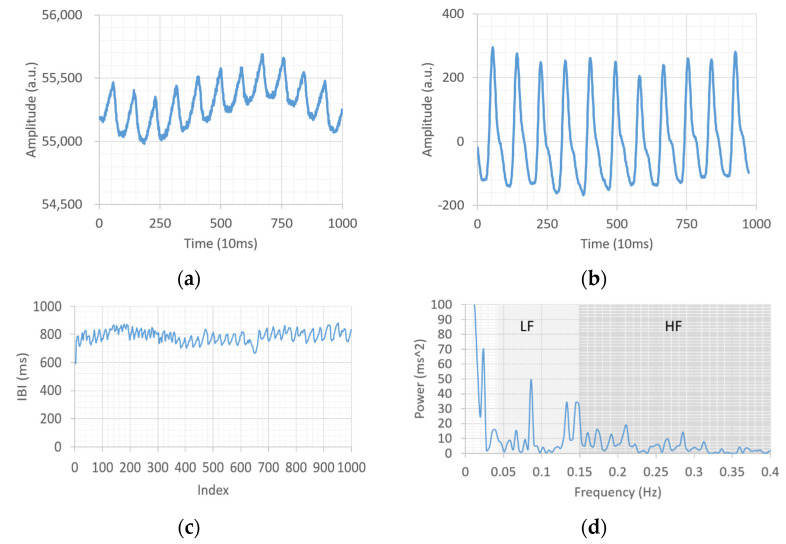
HRV processing stages from PPG signal: (**a**) raw PPG data; (**b**) processed PPG data; (**c**) IBI curve; (**d**) HRV spectrum. a.u. stands for the arbitrary unit.

In this paper, the intermediate node in Figure 1 is implemented as APP software, and the wearable device adopts the smart band provided by a local company, Gadgle Creative Tech. Co, Ltd. (Changhua City, Taiwan), to support long-term PPG measurement after firmware modification. Specifically, the smart band delivers PPG data through Bluetooth and communicates with the data center over a 4G mobile network. Figure 2 shows the smart band used in this paper. In order to ensure the quality of the PPG signal during a game, the smart band uses three LEDs (Figure 2b) to obtain better signal strength and anti-interference ability. Figure 3 presents the PPG raw data collected by a smart band and their signal processing process. In Figure 3b, the peak of the PPG curve represents a heartbeat. The signal processing unit in Figure 1 needs to detect the peak and calculate the time interval between two neighboring peaks, and finally, the IBI sequences (Figure 3c) required to calculate HRV can be obtained. Define the IBI sequence of *n* elements as

(1) $$X=\left\{x_{j}|j=1,2,...,n\right\}$$

where *x_j_* is measured by millisecond (ms). Since the IBI sequence (1) comprises variable time intervals in which two continuous PPG peaks are detected, the HRV calculation unit in APP resamples the received IBI sequences at a fixed time interval [24]. In [25,26,27,28], several alternative approaches are studied to calculate frequency domain parameters without resampling. The fixed-rate resampling methods remain preferred for HRV applications [29,30,31,32]. By denoting the resampling rate as *R*, the resampled IBI sequence with *R* Hz is expressed by

(2) $$\hat{X}=\left\{x_{j}|j=1,2,\ldots ,n+T+D\right\},\;subject\;to\;{log}_{2}^{\left(n+T+D\right)}=M,M\in Z^{+}$$

where *T* is the additional items associated with *R* due to resampling, and *D* stands for the potential padding items to form a resampled sequence with a size of power of 2. The typical value of a padding item is zero. In this paper, the value of *R* is set to 4 Hz following the results reported in [27,28,29,30].

The resampled sequence (2) is then regarded as an input of discrete Fourier transform (DFT)

(3) $$P_{f}=\sum_{k=0}^{\left(n+T+D\right)-1}\hat{x_{k}}\cdot e^{-i2\pi f\frac{k}{\left(n+T+D\right)}}$$

where *P_f_* is measured in a unit of ms^2^. The time complexity of DFT requires O(N^2^), and a fast Fourier transform (FFT) is generally preferred for implementing the frequency domain calculation to achieve a running time of O(Nlog⁡N). Based on (3), the power spectral of HRV is given by

(4) $${HRV}_{f}=\left\{P_{f_{1}},\;P_{f_{2}},\ldots ,P_{f_{n+T+D}}\right\}$$

where $f_{j}$ represents the *j*-th frequency item measured in Hz and can be written as

(5) $$f_{j}=\frac{R}{n+T+D}\times j$$

As shown in Figure 3d, the FFT output at 0.04–0.15 Hz is the LF band in ms^2^, while the frequency segment at 0.15–0.4 Hz is the HF band in ms^2^. Given a series of *P_f_* in the HRV spectrum, LF and HF can be computed respectively as follows:

(6) $$LF=\sum_{f=0.04}^{0.15}P_{f}.\;$$

(7) $$HF=\sum_{f=0.15}^{0.4}P_{f}.\;$$

Furthermore, LF/HF ratio is simply a ratio of LF over HF

(8) $$LHR=\frac{LF}{HF}$$

where LHR > 0. Typically, LHR is used as an indication of sympathovagal balance [33]. A high LHR reflects sympathetic dominance, which generally occurs for fight-or-flight behaviors. On the other hand, a low value of LHR indicates parasympathetic dominance while one conserves energy and engages in tend-and-befriend behaviors [13].

### 2.2. Experiments and Dataset

As discussed in the Introduction section, flow state describes a mental state of optimal user experience. User experience generally comes from comprehensive responses to emotions. Since HRV is known to be related to emotions, its corresponding parameters can be utilized to assess user experience and flow state. This section describes the experiments conducted to measure the flow state of participants in playing video games, and a game fun tracking dataset can be established. In the experiments, the flow state is measured subjectively and objectively: the subjective measurement focuses on a post-game questionnaire based on the percentage system, while the objective measurement monitors HRV parameters such as heart rate (HR), low frequency (LF), and high frequency (HF) during the game. The experiment process is divided into four stages: (1) the participant remains in a sitting position in the resting state and wears the smart band to collect physiological signals for three minutes; (2) the participant starts to play a mobile game for 30 min; (3) the participant stops playing the game, and the physiological measurement conducted by the smart band stops at the same time; and (4) the participant gives a game fun score in the post-game questionnaire. This study utilized a numeric rating scale (NRS) between 0 and 100 to obtain the game fun score. The higher the score, the more game fun is obtained. In order to provide participants a better understanding of the meaning of scores, game fun scores were further divided into four levels: 0–30 represented very low fun; 31–50 represented low fun; 51–70 represented moderate fun; and 71–100 represented high fun. The physiological data collected in stage (2) are divided into 15 segments. Accordingly, each segment contains 2-min IBI signals and is converted to frequency domain HRV parameters.

**Figure 4:**
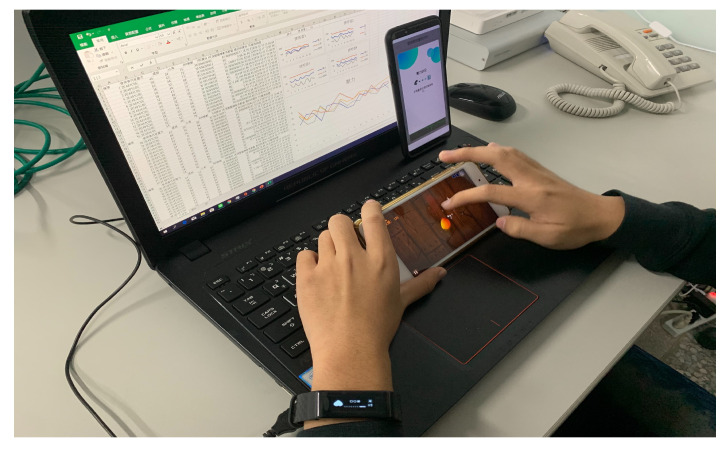
Experiment snapshot.

All participants conducted the experiment in a noise-free room with a temperature of 26 degrees Celsius (i.e., 78.8 degrees Fahrenheit) and an appropriate light. Because HRV is known to correlate with chronic disease and cancer, age and health status are two main characteristics in selecting participants. Eight male and two female healthy participants, aged between 20 and 25 years, were involved in the experiments. The consent of all the participants was obtained after providing them with an explanation of the experiment in terms of purpose and data collection protocol. The mobile games under consideration were Subway Surfers, Fruit Ninja, Tap Tap Music, Tennis Clash, and Push Battle. For each participant, only games that he or she never played before were included in the dataset. One sample was recorded when a participant played a mobile game following the abovementioned four-stage process. The total amount of data collected in the experiments was 36. Figure 4 shows a snapshot captured during the experiment stage (2). From Figure 4, the participant wore a smart band on the left hand to measure heartbeat signals. Meanwhile, an upright smartphone performed an APP to collect heartbeat signals from the smart band continuously. The laptop screen presented the collected APP data for further analysis.

**Figure 5:**
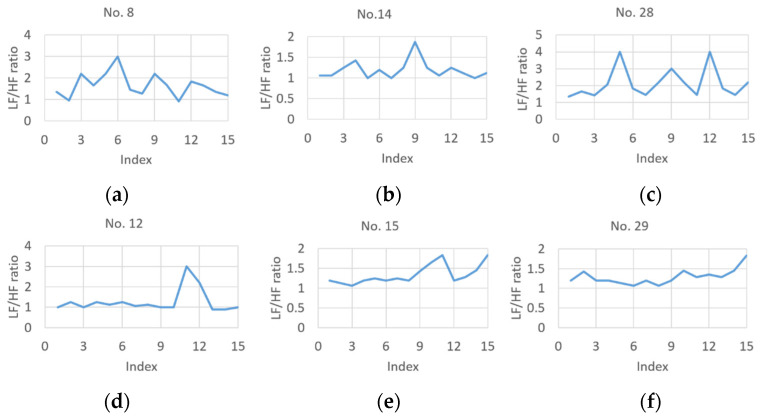
Selected LF/HF ratio curves from dataset: (**a**–**c**) high-score group; (**d**–**f**) low-score group. Each Figure is labeled with its serial number in the experiment.

The collected experiment data constitute the game fun dataset in this paper. In order to observe the potential features among data, the data are divided into two categories according to a game fun score threshold: high-score group (>70); and low-score group (≤70). As a result, the amount of data is 23 and 13 for the high-score and low-score groups, respectively. Table 1 presents the statistics corresponding to two groups. Table 1 shows that for subjective data, the standard deviation (SD) of the high-score group is close to that of the low-score one, and the two groups have different mean values. Figure 5 further shows six selected objective data (i.e., LF/HF ratio): (a)–(c) for the high-score group; and (d)–(f) for the low-score group. The subjective game fun scores are 90, 80, and 75 in sequence for Figure 5a–c, while the scores for Figure 5d–f are 65, 70, and 70, respectively. Compared with the LF/HF ratio curves shown in Figure 5d–f, those of Figure 5a–c have larger fluctuations. In Table 1, compared with the LF/HF ratio curves of the high-score group, the low-score group’s LF/HF ratio curves have nearly the same mean value and achieve a higher SD value since an abrupt change usually occurs with relatively smoother curves.

## 3. Game Fun Prediction and Results

**Figure 6:**
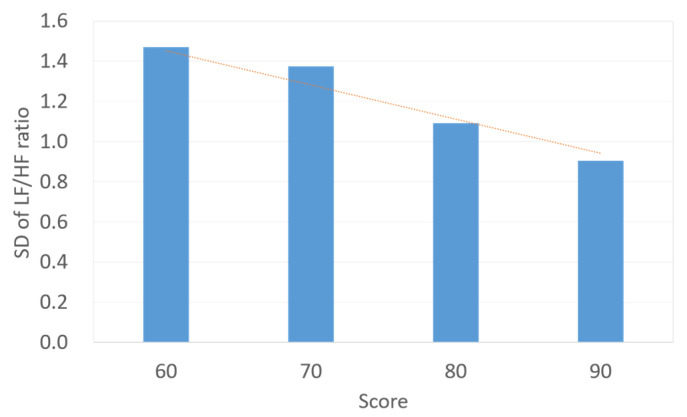
Bar chart and relation (dotted line) between subjective parameter (game fun score) and objective parameter (SD of LF/HF ratio).

**Figure 7:**
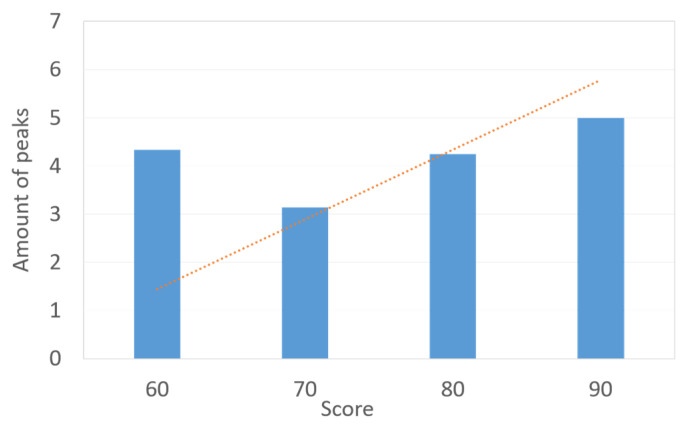
Bar chart and relation (dotted line) between subjective parameter (game fun score) and objective parameter (amount of peaks).

**Figure 8:**
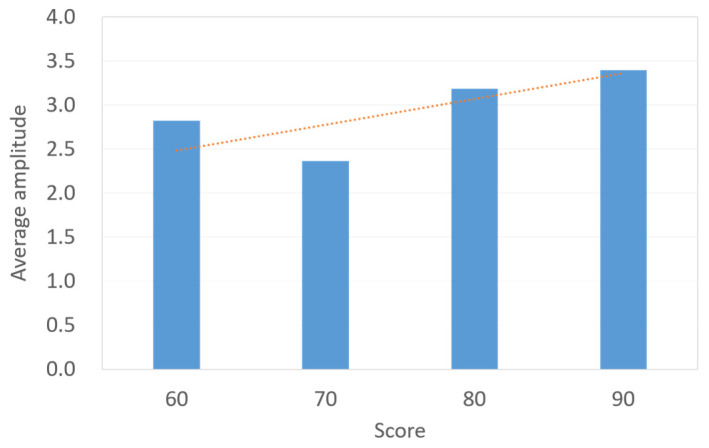
Bar chart and relation (dotted line) between subjective parameter (game fun score) and objective parameter (average amplitude of the peak).

According to the analysis of the dataset collected as described in Section 2, it is seen that (1) for an average game fun score, a difference between the high-score group and low-score group exists, and (2) compared with the low-score group, the high-score group has larger fluctuations in LF/HF ratio curve. This paper aims to use the features of the objective LF/HF ratio to predict the subjective game fun scores. For the dataset with a sample size of 36, Figure 6 presents the relation between the game fun score and the SD of the LF/HF ratio. In Figure 6, the SD values gradually decrease as the score exceeds 60. It is conservative to assume that the SD of the LF/HF ratio is inversely proportional to the subjective score. Figure 7 illustrates data distribution between game fun scores and the number of peaks for LF/HF ratio curves. Since a wave can be regarded as a combination of a peak and its neighboring valley, the amount of peaks is considered in this study to represent the number of waves. In Figure 7, more peaks are observed for higher scores beyond 70, and the number of peaks is proportional to the subjective score. Figure 8 shows the relation between the game fun score and another objective parameter relating to curve oscillation, namely, the average amplitude of the LF/HF ratio curve. The average amplitude slowly declines as the score decreases for the high-score group.

Although the sample size in the dataset is relatively small, it can be seen in Figure 6 that three HRV indicators correlate with game fun scores for the high-score group. The player who experiences greater game fun is more likely to be measured with a smaller SD, more number of peaks, and larger amplitude in terms of the LF/HF ratio curve. On the other hand, Figure 7 and Figure 8 show a correlation discontinuity between the high-score and low-score groups. By inspecting the data distribution in the dataset, the data of the low-score group are more scattered, while the data from the high-score group tend to be concentrated and stable. These data features may indicate that the low-score players’ diverse emotional responses, such as boredom, frustration, and impatience, cause inconsistent HRV responses.

Based on the abovementioned relations between HRV indicators and subjective scores for the high-score group, a linear model to predict game fun score *G*′ can be expressed as

(9) $$G^{\prime }=\alpha \times \frac{1}{LHSD}+\beta \times LHP+\gamma \times LHA+\varepsilon$$

Table 2 lists the definition of all notations in (9). Estimating parameters of (9) is known as a regression problem. In order to observe the performance effect on correlation discontinuity, two prediction models were developed: Model 1 selects the dataset of the high-score group as the training data for (9), and its optimal parameter vector {α, β, γ, ε} is {0.21, 1.76, −0.46, 72.59} with *R*^2^ = 0.20. In addition, Model 2 uses the whole dataset to build a prediction model based on (9) for the low-score and high-score groups. The parameter vector {α, β, γ, ε} of Model 2 is {3.10, 2.03, 2.29, 54.27} with *R*^2^ = 0.21. Although building the respective training data for Model 1 and Model 2 is intuitive, the relation discontinuity can impede group differentiation. As a result, it can be difficult to apply the proper prediction model for a sample without a group label. As shown in Figure 6, the SD of LF/HF ratios has a linear continuity across two groups and can be adopted to realize the group differentiation in this study. By inspecting data in Figure 6, a sample is regarded as a low-score group as the SD value is smaller than 1.47. Otherwise, the sample belongs to the high-score group.

Table 3 presents the results of prediction errors in terms of MAE and RMSE to evaluate the prediction performance. MAE and RMSE can be defined as

(10) $$\left\{\begin{array}{c} \mathrm{MAE}=\frac{\sum_{i=1}^{N}\left|G_{i}^{\prime }-G_{i}\right|}{N},\\ \mathrm{RMSE}=\sqrt{\frac{\sum_{i=1}^{N}{\left(G_{i}^{\prime }-G_{i}\right)}^{2}}{N}} \end{array}\right.$$

where *N* is the number of data, and $G_{i}$ represents a subjective game fun score of player I; $G_{i}^{\prime }$ stands for the predicted game fun score for player i. Currently, the value of *N* is 21 and 36 for Model 1 and Model 2, respectively. As expected, Model 1 has lower MAE and RMSE values than Model 2. For Model 1, the MAE of 4.16 is obtained, and the prediction error rate can be further calculated as (4.16/100) × 100% = 4.16% in the case that the game fun score is ranged from 0 to 100. In Model 2, the high-score group has lower MAE and RMSE values than the low-score group. Consequently, the MAE of 7.22 is attained for all participants.

Table 4 further gives an ablation study for the prediction model with varied HRV indicators. While only one indicator is considered in predicting game fun scores, LHP has the smallest MSE and RMSE values for both Model 1 and Model 2. In the case of two indicators, the best combination in Model 1 is LHSD and LHA, while a combination of LHSD and LHP obtains the smallest prediction errors in Model 2. For both Model 1 and Model 2, it can be observed that the prediction errors are decreased as the number of indicators is increased. Consequently, the prediction model with three indicators outperforms the one-indicator and two-indicator models.

In addition to scores, game fun classes or stars can be common rating tools in the game industry. In this study, the scores are evenly divided into 10 classes in units of 10 and 5 classes in units of 20, respectively. The prediction is correct, while the predicted and subjective scores are in the same class. The compared work refers to a trailer-based method [34]. In the trailer-based method, images and text descriptions associated with trailers of video games contribute to predicting game fun scores. Based on a deep learning model combining convolution and long short-term memory (LSTM) networks, the trailer-based method predicts one class from 10 game fun classes. Although both the trailer-based method and the proposed HRV-based method follow a similar 10-class prediction, the dataset between them differs in terms of games and subjective scores. Consequently, the trailer-based method is considered only for reference purposes.

Table 5 gives the prediction accuracy results. For the 10-class rating system, the proposed Model 1 achieves a prediction accuracy of 85.2%, while an accuracy of 100% is attained for the 5-class system. When both low-score and high-score groups are considered in Model 2, the prediction accuracies of 70.6% and 100% are obtained for the 10-class and 5-class systems, respectively. The trailer-based method reports an optimal accuracy of 70.5% for the 10-class rating system [34]. Although datasets between the two compared methods differ, the HRV-based method can provide real-time feedback during the game, while the trailer-based method better fits pre-game recommendations.

## 4. Discussions

This study aims to observe if the subjective user experience, typically measured by numeric rating scales, can be assessed using objective physiological signals. This paper utilizes the smart band with PPG sensors to measure players’ HRV signals during the game while collecting their post-game fun scores. Based on the correlation between game fun scores and physiological data, a linear model is further derived to predict the game fun score using objective features extracted from continuous LF/HF ratios. According to the analytical results, the proposed prediction model for the high-score group can obtain an MAE rate of 4.16% and an RMSE rate of 5.07% in the percentage score system. In addition, the classification accuracies of 85.2% and 100% are attained for the 10-class and 5-class score systems, respectively.

It can be further observed from the results that (1) the HRV features show linear distributions for the high-score group. This observation implies the potential measurement of flow state via HRV-based signals, and (2) the non-continuous data relation exists between the high-score and low-score groups. Furthermore, HRV data of the low-score group are characterized by a scattered distribution. The observations of the low-score group indicate that the players less interested in the target game may express inconsistent emotional responses. These responses regarding LF/HF ratio-related parameters can be partially similar to those of the high-score group.

The main contributions of this study can be summarized in the theory and application domains. In the theory domain, three HRV-derived indicators are shown to be highly related to high game fun scores. This finding and its corresponding prediction model provide a potential method to assess the flow state. It is also observed that data relation is not continuous between low-score and high-score groups. This discontinuity suggests an open issue to exploit the appropriate indicator(s) and to build a unified prediction model for both groups accordingly. In the application domain, objective game fun assessment and wearable-based measurement are advantageous to improve user experience. For instance, the game content (e.g., levels or modes) can be altered in accordance with the individual difference.

The limitations of this study included a small sample size, a limited range of ages, an imbalance of genders, and the selection of mobile games. First, the sample size was relatively small. A more rigorous experiment design would be required to recruit more samples to determine whether the results are generalizable. Second, this study selected young adults as participants to prevent the negative effects induced by chronic diseases on HRV data. The limited range of ages, however, limited the generalizability of our findings. The imbalance of genders in the dataset also puts a limit on generalizability. Third, the characteristics of selected mobile games in this study were easy to play with different levels or various modes; thus, the collected game fun scores were at least 60. The future works of this paper include extending the game fun dataset, involving all available HRV parameters in the dataset, and studying the correlation between subjective and objective data for the low-score group. The available HRV parameters consist of time domain variables, such as the standard deviation of IBIs and root mean square of successive differences (RMSSD) and frequency domain variables, such as LF peak, HF peak, and total power. In extending the dataset of the low-score group for further analysis, public reviews or critics are collected as references for game selection.

## Figures and Tables

**Table 1:** Statistical results for high-score and low-score groups.

Group	Amount	Subjective (Game Fun Score)		Objective (LF/HF Ratio)	
		Mean	SD	Mean	SD
High-score	23	81.00	5.54	2.10	0.70
Low-score	13	65.00	4.80	2.11	0.82

**Table 2:** Notations of Equation (9).

Notation	Definition
LHSD	SD of LF/HF ratios
LHP	Amount of peaks for LF/HF ratio curve
LHA	Average amplitude of LF/HF ratio curve
α, β, γ	Linear parameters
ε	Prediction error compensation parameter

**Table 3:** Prediction error statistics.

Model	MAE	RMSE
Model 1	4.16	5.07
Model 2 (Low-score)	8.03	9.57
Model 2 (High-score)	6.71	8.22
Model 2 (All)	7.22	8.76

**Table 4:** Prediction error comparisons among various combinations of HRV indicators.

	Model 1		Model 2 (All)	
Indicators	MSE	RMSE	MSE	RMSE
LHSD	4.74	5.54	8.18	9.78
LHA	5.03	5.66	7.96	9.62
LHP	4.59	5.39	7.71	9.44
LHSD + LHA	4.35	5.30	7.59	9.21
LHSD + LHP	4.59	5.38	7.46	9.00
LHA + LHP	4.43	5.29	7.69	9.43
LHSD + LHA + LHP	4.16	5.07	7.22	8.76

**Table 5:** Classification accuracy comparisons among trailer-based and HRV-based methods.

Method	10 Classes	5 Classes
Trailer-based [34]	70.5%	−
HRV-based (Model 1)	85.2%	100%
HRV-based (Model 2)	70.6%	100%

## Data Availability

The original contributions presented in this study are included in the article, and further inquiries can be directed to the corresponding author.
